# Automated Home Oxygen Delivery for Patients with COPD and Respiratory Failure: A New Approach

**DOI:** 10.3390/s20041178

**Published:** 2020-02-20

**Authors:** Daniel Sanchez-Morillo, Pilar Muñoz-Zara, Alejandro Lara-Doña, Antonio Leon-Jimenez

**Affiliations:** 1 Biomedical Research and Innovation Institute of Cadiz (INiBICA), 11009 Cadiz, Spain; 2 Biomedical Engineering and Telemedicine Research Group, Department of Automation Engineering, Electronics and Computer Architecture and Networks, University of Cadiz, 11519 Puerto Real, Cadiz, Spain; 3 Pulmonology, Allergy and Thoracic Surgery Unit, Puerta del Mar University Hospital, 11009 Cadiz, Spain

**Keywords:** COPD, oxygen concentrator, oxygen therapy, automatic oxygen concentrator, physical activity, machine learning, respiratory medicine, portable oxygen concentrator, oxygen delivery

## Abstract

Long-term oxygen therapy (LTOT) has become standard care for the treatment of patients with chronic obstructive pulmonary disease (COPD) and other severe hypoxemic lung diseases. The use of new portable O_2_ concentrators (POC) in LTOT is being expanded. However, the issue of oxygen titration is not always properly addressed, since POCs rely on proper use by patients. The robustness of algorithms and the limited reliability of current oximetry sensors are hindering the effectiveness of new approaches to closed-loop POCs based on the feedback of blood oxygen saturation. In this study, a novel intelligent portable oxygen concentrator (iPOC) is described. The presented iPOC is capable of adjusting the O_2_ flow automatically by real-time classifying the intensity of a patient’s physical activity (PA). It was designed with a group of patients with COPD and stable chronic respiratory failure. The technical pilot test showed a weighted accuracy of 91.1% in updating the O_2_ flow automatically according to medical prescriptions, and a general improvement in oxygenation compared to conventional POCs. In addition, the usability achieved was high, which indicated a significant degree of user satisfaction. This iPOC may have important benefits, including improved oxygenation, increased compliance with therapy recommendations, and the promotion of PA.

## 1. Introduction

Oxygen is a substantial element in the sustenance of human life. Of the hundreds of tasks that oxygen performs in the human body, two stand out for their importance: detoxification and energy production. However, some diseases affect the ability of the lungs to perform the gas exchange necessary to incorporate oxygen into the bloodstream and to release carbon dioxide. In many situations, extra oxygen is needed when the respiratory system cannot maintain an adequate pulmonary exchange of physiological gases. This therapeutic use of supplemental oxygen is defined as oxygen therapy and it aims at increasing the inspired oxygen fraction (FiO_2_). Therefore, when medically prescribed, oxygen is a drug. Oxygen therapy is an established treatment, and it continues to be one of the most important measures in the management patients with progressing chronic respiratory disease. In this case, the main objective of oxygen therapy is to improve tissue oxygenation and to correct the severe hypoxemia that these patients usually present with in the advanced stages of the disease [1,2]. The goal is to maintain oxygenation levels above the range of respiratory failure, defined by an O_2_ blood partial pressure [PaO_2_] > 60 mmHg, and an oxygen saturation measured by pulse oximetry [SpO2] > 90%.

Currently, clinical applications for the use of oxygen have extended beyond the hospital setting. When patients receive oxygen supplementation at home, the therapy is referred to as home oxygen therapy (HOT). HOT provided as long-term oxygen therapy (LTOT), that is, used on a daily basis and at least by 15 h per day, is recommended by current treatment guidelines since it has been shown to be effective in increasing survival in patients with chronic obstructive pulmonary disease (COPD) and respiratory failure [3,4,5,6]. In addition, ambulatory oxygen therapy improves physical performance in patients with COPD [7].

Despite all these benefits of HOT for patients with respiratory failure, evidence that supports the prescription of HOT in other chronic conditions associated with hypoxemia is limited [8]. While supplemental oxygen is valuable in clinical situations such as those aforementioned, the inappropriate use of this therapy can be detrimental. Hypoxemia is defined as the decrease in PaO_2_ below the normal limits, variable for the subject’s age (normal PaO_2_ ranges from 80 to 100 mmHg [9]). There is evidence that both hypoxemia and hyperoxemia, which results from exposure to excessive O_2_ flows for a prolonged period of time, can have serious consequences for patients with acute and chronic respiratory failure [10,11]. Although the risks for hypoxemia are well known [12], there is growing evidence that excessive oxygen flow can be potentially harmful. In this regard, hyperoxemia has been associated with increased hospital mortality among patients admitted to intensive care units (ICUs) following cardiac arrest resuscitation [13]. In this regard, hyperoxemia may be especially problematic in patients with COPD in the acute phase of exacerbation, because of its association with hypercapnia [14,15] and its potential to mask the onset of a worsening in lung function [10]. In addition, toxicity caused by hyperoxemia in some patients with COPD receiving LTOT has received attention from researchers [16].

Notwithstanding this, all the scientific evidence supports the idea that the proper use of supplemental oxygen therapy is an important factor that can positively influence clinical outcomes in patients with respiratory failure and severe hypoxemia [17].

The administration of LTOT requires devoted delivery devices. The source of oxygen and the equipment for its administration, which will depend on the patient’s profile, his/her movability, the flow required, the time needed for oxygen therapy sessions, and above all, the proper correction of SpO_2_ both at rest and during sleep or effort are also important factors [18].

Until recently, the most common way to deliver LTOT to patients has been by using static sources, such as stationary oxygen concentrators or high-pressure cylinders. The shortcoming of such devices is that they prevent the patient from wandering or leaving home while therapy is being received. The heightened mobility and physical activity of these patients have resulted in the need for smaller, lighter, and more autonomous portable oxygen devices [19]. For those reasons, a new generation of portable, lightweight devices has emerged in recent years. These devices, known as portable oxygen concentrators (POCs), have sufficient autonomy to enable the patient to live an active life outside the home. Unlike classical gaseous or liquid oxygen devices, POCs produce their own oxygen by removing nitrogen from atmospheric air. Before it goes into the concentrator through the inlet filter, air is composed of 80% nitrogen and 20% oxygen. Firstly, the POC compresses the oxygen using a compressor. The compressed air moves to a sieve bed of filters that separate the nitrogen from the oxygen. Then, the oxygen, now at around 90%–95% purity, is stored in a product tank within the device and is delivered to the patient via a delivery device (i.e., a nasal cannula).

POCs can deliver oxygen via continuous flow or pulse flow. Continuous flow units put out a specific adjustable dose measured in litres per minute. Pulse units pulse air through a cannula with each breath and their output is determined by the size of the individual pulse (millilitres per pulse) and the patient’s respiratory rate.

It is accepted that the oxygen flow, normally delivered in fixed doses to patients in oxygen therapy, is not always optimized [19]. In fact, the oxygen requirements of patients under LTOT vary depending on the type, intensity and duration of the physical activity that is being carried out. Low blood oxygen levels may cause short-term symptoms, such as dyspnoea, and physio-pathological organic changes, such as tachycardia, increased respiratory rate, arterial hypertension and, in the long term, serious problems such as pulmonary hypertension and cor pulmonale, among others. On the other hand, excessive levels of blood oxygen can cause hypercapnic encephalopathy in some patients. In this regard, the patients receiving LTOT are generally instructed to adjust the oxygen flow according to the activities of daily living. Oxygen flow is therefore routinely targeted to maintaining the desired oxygenation range. However, this task places a burden on patients that often affects adherence to therapy, and the existing methods of oxygen delivery may not be sufficient when the patient’s activity, and therefore the demand of oxygen, increases [20]. It has been reported that patients with COPD and moderate hypoxemia have frequent and eventually significant desaturations during activities of daily living and at night [21].

Adapting oxygen therapy to dynamic patients’ needs appears to be a key challenge. Among the primary goals of the dynamic and adaptive oxygen flow adjustment are: (a) the optimization of therapy and safety by minimizing the number of desaturation episodes, preventing periods of hyperoxia and hyperoxia [12]; (b) the customization of the oxygen flow to the individual needs of patients; and (c) oxygen consumption optimization.

In traditional flow oxygen delivery, the titration of oxygen therapy is generally performed manually by selecting the level of oxygen flow [22]. In LTOT, this manual adjustment of oxygen flow is carried out by patients themselves to meet their changing needs. The current POCs have a manual flow regulator that is adjusted by prescription to a specific level of flow measured in litres or pulses. Existing flow regulators modify the flow mechanically, by manipulating a valve, or electronically, by means of a keypad integrated into the device. However, the manually continuous adjustment of the oxygen flow rate is a time-consuming task that requires experienced and trained patients [23], and this can lead to the improper use of the device and consequently to a poor quality flow adjustment during the changing daily activities, which is usually linked to unintended delays and periods of desaturation [24,25]. Given the advanced average age of patients with COPD under LTOT [26], the probability of inadequate use of the device is significant.

Very recently, the abovementioned limitations led to the search for new physiological closed-loop devices (PCLC) that timely adjust oxygen flow rates to the needs of patient automatically [22]. PCLC medical devices use one or several physiological sensors to manipulate a physiological variable autonomously according to the guidelines given by clinicians [27]. Exponents of PCLC in respiratory medicine are the novel intelligent portable oxygen concentrators. Most of the published articles on technologies for PCLC in oxygen therapy for adults are from post-2010, indicating that these devices are a relatively new field in the respiratory speciality. These devices may potentially optimize oxygen therapy, reduce the workload of health professionals, minimise medical error, shorten health care costs, and decrease mortality and morbidity [28,29].

**Figure 1:**
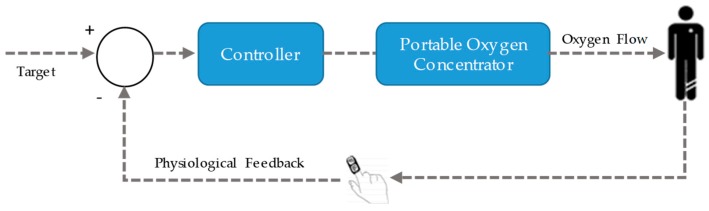
Common architecture of mostly proposed closed-loop portable oxygen concentrators.

Intelligent POCs include three main components: a system for monitoring the patient’s oxygenation, an algorithm to estimate the O_2_ flow settings to achieve the targeted oxygenation level, and an O_2_ source [20]. Figure 1 depicts the global architecture of a PCLC oxygen therapy device.

In the most common approach, the process variable is selected to be SpO_2_, since it is a non-invasive measure that does not require calibration. A recent systematic review reported that SpO_2_ (pulse oximeter) was used in 100% of studies on PCLC systems for automatic oxygen delivery in patients with COPD [25]. Concerning the algorithms, the review concluded that continuous control by applying conventional classical proportional-integral-derivative (PID), proportional-integral (PI), and rule-based controllers have been proposed in the context of LTOT systems [30,31,32,33]. In these studies, the most significant findings were related to the shortening of hypoxemia and hyperoxemia episodes when PCLCs were used. However, a challenge in these algorithms is related to stability and robustness, associated with the system capacity to discriminate between real abnormal oxygenation events and ghost episodes caused by motion artefacts or a poor-quality oximetry signal. This major challenge has not been overcome, and, at present, there are only a few commercial systems available (i.e., Optisat AccuO2^®^ [34], the O2 Flow Regulator^®^ [33], and the FreeO2^®^ [19]). The existing systems require the patients to be continuously connected to a pulse oximeter, which becomes the primary source of information for the algorithm. Current pulse oximetry technology is marked by the instability of the sensor relating to movements or by the physiological delay in the measurements, which may reduce its clinical effectiveness. Additionally, this technology presents very limited effectiveness in assessing hyperoxia and promptly detecting respiratory depression [22]. Moreover, pulse oximeters are known to be inaccurate in conditions that decrease arterial blood perfusion or cause the presence of elevated concentrations of carboxyhaemoglobin (CoHb) and methaemoglobin (metHb).

As a consequence, the development of innovative physiological sensors and predictors of oxygen desaturations to enable autonomous therapy and support its clinical validity is still an on-going challenge [35].

In addition to technological matters, patient perceptions on usability and adequacy of POCs is an issue. In a recent study, 51% of the patients under LTOT consulted reported oxygen problems related to equipment malfunction, a lack of physically manageable portable systems, and a lack of portable systems with high flow rates [36]. In that study, 44% of respondents referred to the limitations in activities outside the home imposed by inadequate portable oxygen systems.

In summary, despite their potential and the existing end-users (patients) demand, PCLC oxygen therapy devices are scarcely found in real clinical applications. The robustness of control algorithms, the limited reliability of sensors, safety and usability issues, among others, can underlie this lack of clinical implementation [25].

In this work, we propose an alternative approach that enables a POC to be transformed into an intelligent POC (iPOC) to adjust the oxygen flow automatically in patients with COPD and respiratory failure that receive LTOT. The proposed system is based on the automatic classification of the intensity of the patient’s physical activity and can adjust the oxygen flow to individual real-time needs autonomously. It is a transdisciplinary work, rooted in the field of respiratory medicine, with contributions from electronics, control theory, computer science and artificial intelligence.

An external portable electronic system was designed and integrated into a commercially available POC. The system comprised two units: 1) a sensor unit attached to the patient, that classifies the physical activity in real-time; and 2) a receiver unit, interfaced to the POC, that adjusts the oxygen flow according to the input from the sensor unit automatically. The algorithm for the automatic recognition of physical activity was trained and validated using machine learning techniques. A circuit was designed to gather data for personalizing models and to evaluate the system performance in a group of patients with COPD and respiratory failure receiving LTOT.

The rest of the paper is organised as follows. Section 2 details the participants, materials, devices and methodology applied to develop the iPOC system and to conduct the experiments. In Section 3, the results achieved are presented and discussed. Finally, Section 4 captures the conclusions and future works.

## 2. Materials and Methods

### 2.1. System Architecture and Working Principle

**Figure 2:**
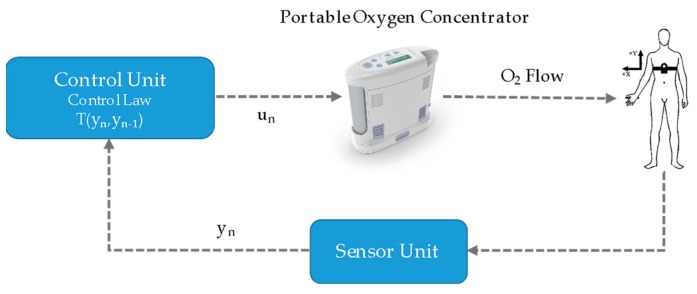
The architecture of the proposed intelligent closed-loop portable oxygen concentrator (*iPOC*).

Figure 2 shows the general outline of the proposed iPOC. The closed-loop control was implemented using a SISO (Single Input - Single Output) controller [37]. The control law is defined by a lookup-table controller. The oxygen flow level (output) corresponding to each state of physical activity intensity (y_n_) was defined by the pulmonologists and personalised to the needs of each patient according to conventional clinical assessment procedures [38]. The system automatically identifies the intensity of the patient’s physical activity by classifying it into one of a 3-class scheme (sedentary, light, and moderate). The intensity of physical activity detected in real-time is communicated to a control unit connected to the POC, which is responsible for adapting the oxygen flow to the level previously calibrated by the physician for each situation. Therefore, when an increase in the intensity of the patient’s physical activity is detected, the system manages the automatic increase in oxygen flow. Conversely, when the patient lowers the intensity of the physical activity, the device orders the flow to be decreased to the predetermined level for that new condition. In addition, the automatic dosing device can be deactivated by switching the system to the conventional manual mode. The proposed system comprises a unit for estimating the intensity of the patient’s physical activity (sensor unit) and unit for controlling the oxygen supplied flow (control unit). These units where designed iteratively using a patient-centred approach. Each of these elements will be described in more detail in the next subsections.

#### 2.1.1. Sensor Unit

The sensor unit is a portable module placed on the patient’s chest. Accelerometer- and gyroscope-based physical monitoring systems have been shown to be able to discriminate between different daily activities [39,40,41,42]. Each of these physical activities leads to a different level of energy expenditure, and in patients under LTOT, to different oxygen flow requirements. For the estimation of the intensity of physical activity, an inertial measurement unit (IMU) based on MEMs technology was used. This IMU included a three-axis accelerometer, a triple-axis gyroscope, and a barometer. The signals acquired by the IMU were processed using a microcontroller (MCU) and digital signal processing techniques to extract features useful for discrimination. A classification model, trained and validated using machine learning algorithms, automatically classified the intensity of the activity and transmitted this information wirelessly to the control unit.

**Figure 3:**
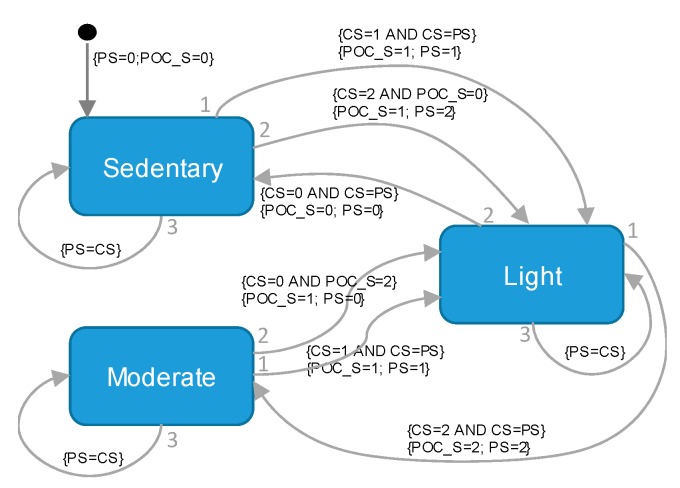
The Mealy finite-state machine included as output layer to provide a supplementary safety mechanism in the automatic delivery of oxygen flow. PS: Previous state; POC_S: Portable Oxygen Concentrator State; CS: Current state.

The sensor unit incorporated a Mealy finite-state machine (FSM) after the classification stage as a safety mechanism (Figure 3) to prevent abrupt changes in the flow of oxygen (e.g., change of state from a sedentary level to a moderate one or vice versa).

**Figure 4:**
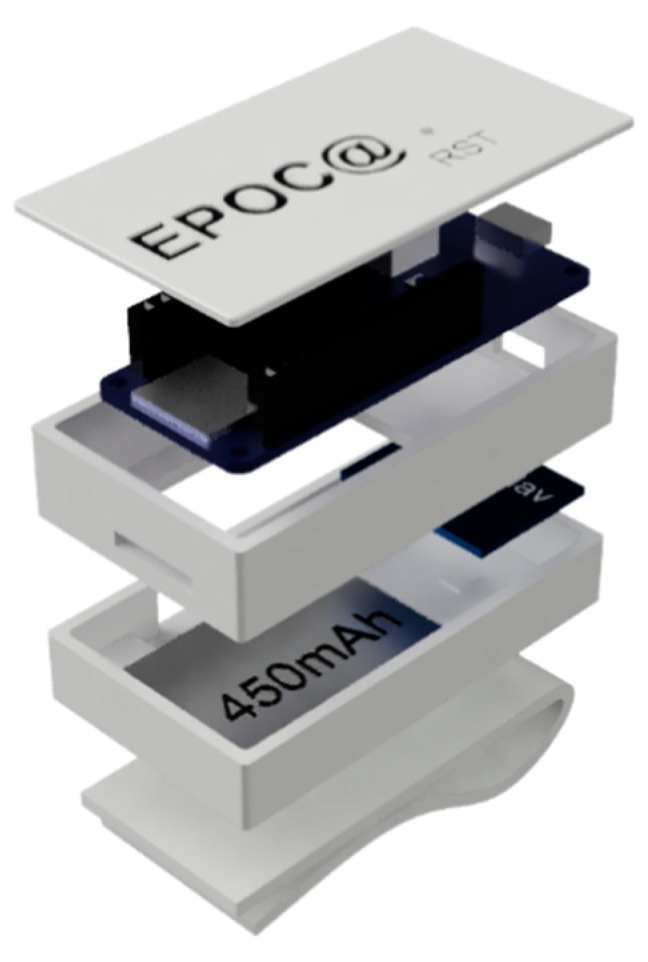
The sensor unit implemented for data logging in the first design stage.

In order to conduct the research, two prototypes of the sensor unit were developed, each with different purposes and requirements. The first prototype was designed to perform raw-data collection using a µSD card during the training and internal validation of the machine learning models (Figure 4). It was equipped with a SAM-D21 (ARM M0+ 32bits, 256KB Flash, 32KB RAM) microcontroller. An RGB LED was added to show the device state (e.g., error state, standing state, starting data recording state and recording data state).

**Figure 5:**
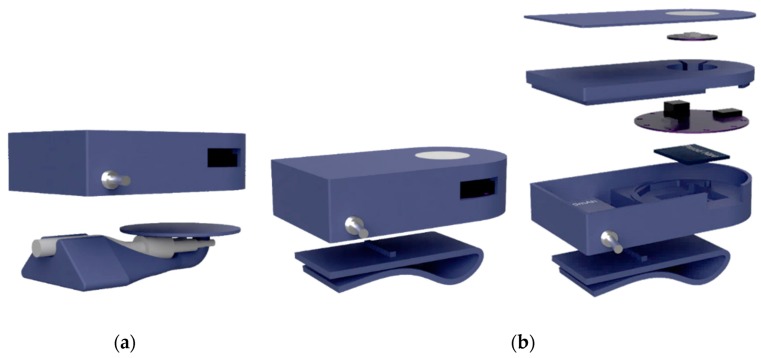
Three-dimensional design of the embodiment of the final prototype of the sensor unit including: (**a**) an adapter to the nasal cannula; (**b**) an adapter for a chest-band.

The second prototype was implemented to host the final trained classification model including the FSM. It featured an ARM M0 (32bits, 128KB Flash and 24KB RAM) and wireless Bluetooth Low Energy (BLE) communications. Both prototypes were equipped with the 9-axis MEMS sensor LSM9DS1, which includes 3 digital acceleration channels (±2/±4/±8/±16 g linear acceleration full scale), 3 angular rate channels (±245/±500/±2000 dps angular rate full scale), SPI/I2C serial interfaces, 16-bit data output and programmable interrupt generators. In addition, an LPS25HB MEMS piezo-resistive pressure sensor was used. The PS25HB barometer measurement range is from 260 to 1260 hPa. Pressure sensor information showed poor sensitivity for real-time classification purposes and was not used in the study. Both PCBs incorporated a 600 mAh lithium polymer (LiPo) battery, management connectors and circuits. The housing of the final prototype was designed with a double patient fixation option: nasal cannula tube fixation (Figure 5a) and thoracic elastic band fixation (Figure 5b).

The sensor unit draws around 10 mA with 60 mA peaks when Bluetooth communications are used. In the worst scenario, considering an average consumption of 30 mA and the 600 mAh battery, the unit performs monitoring for about 14 h using the 600 mAh battery. This operating time is significantly longer than that of the oxygen concentrator, which ranges from 2 to 5 h.

**Figure 6:**
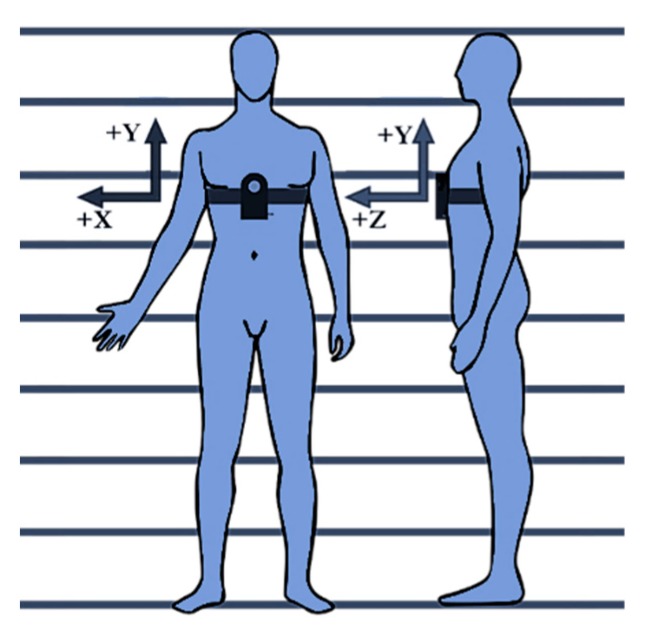
Preferred placement of the sensor unit on the subject’s body.

The sensor unit was placed in the chest of the user, just at the end of the sternum. It has been reported that this location generates fewer motion artefacts due to movements compared to systems placed on the wrist, ankle or the belt [43]. The unit was mounted to ensure that the Y-axis of the IMU pointed at the head of the subject, and the Z-axis in the walking course. (Figure 6).

#### 2.1.2. Control Unit

**Figure 7:**
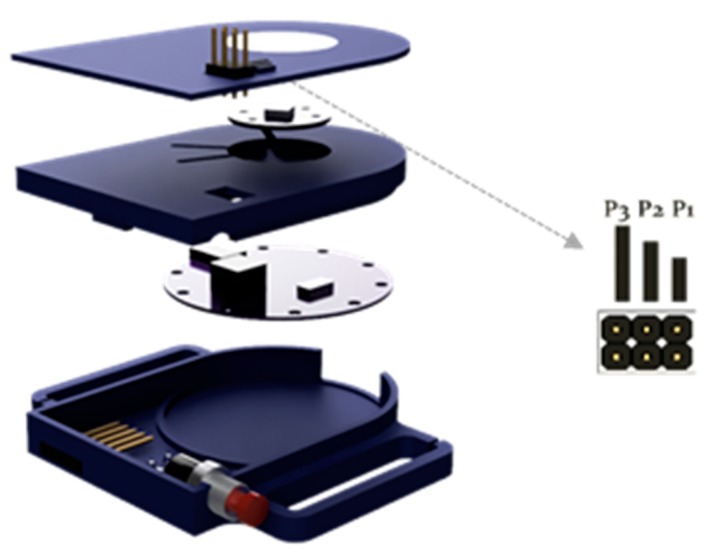
The control unit implemented for communication with the sensor unit and for controlling the portable oxygen concentrator. Header jumpers were used to personalize oxygen therapy settings and push button to switch the portable oxygen concentrator operating mode (manual or automatic).

The control unit receives the estimate of the intensity of the patient’s physical activity and is responsible for adjusting the oxygen flow supplied to the patient according to the instructions established during the process of tuning of the therapy. The control unit includes an MCU and wireless communication capabilities, and interfaces directly with the POC. It was equipped with an ARM M0 (32bits, 128KB Flash and 24KB RAM), wireless BLE communications, an RGB LED, and a push-button to activate the automatic mode in the POC (Figure 7). The unit was directly powered from the POC.

The control of the concentrator by the control unit was carried out by simulating physical pulses to the increase and decrease buttons in the touch keyboard of the POC. As a consequence, the interface between the control unit and the POC was implemented using a 5-pin connector that enabled the power supply of the unit and the switching of the signals to increase or decrease the flow. In addition, a 6-pin male connector was included to enable the personalisation of different oxygen levels settings using header jumpers (see Table 1 and Section 2.1.2). As an example, the setting number 2 should be selected (jumper P2 on) for a patient who requires, at 15 breaths per minute, a bolus volume of 12 mL at rest, of 36 mL while doing light intensity activity, and of 60 mL when doing moderate intensity activity.

The control unit presents the same power consumption as the sensor unit. It is powered from the POC and causes a battery drain of 214 mAh, what supposes a decrease of about 12 min in the concentrator operating time.

#### 2.1.3. Portable Oxygen Concentrator

**Figure 8:**
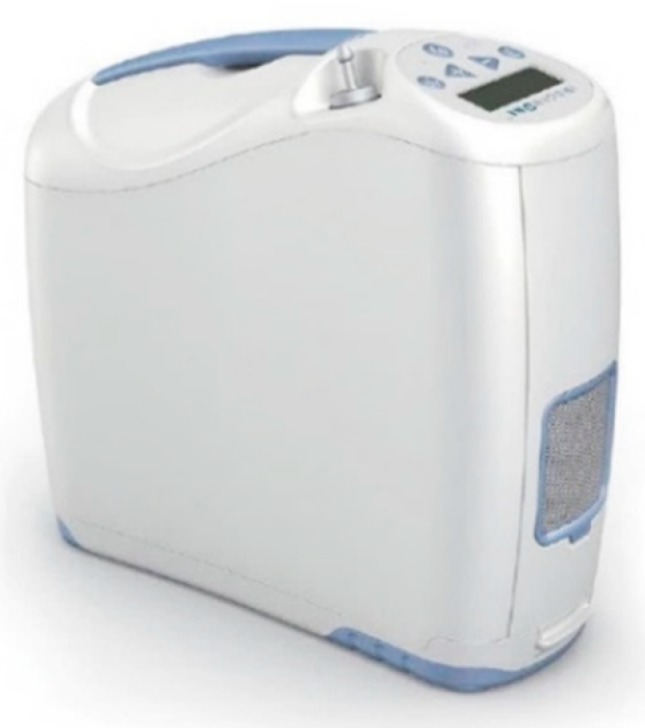
Inogen One G2 Portable Oxygen Concentrator used in the study.

Inogen One G2 POC was used in the study (Figure 8). The Inogen One G2 delivers up to 900 mL/min of 90% oxygen and supports pulsed dose delivery [44]. This concentrator has five levels for the adjustment of the oxygen needs. The oxygen dose applied to each patient in each situation depended on the previous titration by the specialist.

In general, this POC delivers 12 mL per bolus per flow setting at 15 breaths per minute (180 mL/min per-flow setting). Table 2 summarizes the bolus volumes delivered at reference environmental conditions. Slower breathing patients will receive larger boluses, and faster breathing patients will receive smaller boluses.

#### 2.1.4. Communication Protocol

The units were programmed using the Arduino Integrated Development Environment (IDE). The bidirectional communication between the sensor unit and the control unit takes place using low-latency Bluetooth 4.0 (BLE) with 128-bit AES CCM (counter with cypher block chaining message authentication code CBC-MAC) encryption/decryption without pairing. The messages are sent encrypted with the serial number of the sending microcontroller and it is the receiver that uses the serial number to decrypt the messages. The wireless control can be authenticated through toasts for the confirmation of the desired operations and also tracked through timestamps. BLE remains in sleep mode at all times, except when participating in a data exchange, which reduces overall energy consumption. In this manner, the sensor unit only sends messages when there is a change in physical activity intensity.

### 2.2. Study Design and Participants

A total of 18 volunteers (Table 3) were recruited for this study at the Pneumology, Allergy, and Thoracic Surgery Unit of the University Hospital Puerta del Mar de Cadiz (Spain) (14 male, average age 66.9 ± 12.8 years, range 60–93 years, average body mass index (BMI) 27.00 ± 3.6 kg/m^2^). The participants selected had a diagnosis of COPD and stable chronic respiratory failure, and were receiving LTOT and using a POC.

Exclusion criteria included any organic comorbidity that could cause or contribute to exertional dyspnoea that would hinder the realization of the circuit (cardiovascular diseases, metabolic or other associated respiratory diseases), COPD exacerbation within the six weeks before the enrolment or any disease that could limit the physical activity of the patient (e.g., neuromuscular or skeletal diseases). The general fragility (i.e., difficulty in walking or lack of autonomy) that could substantially prevent the patient’s participation in the study was also considered an exclusion criterion, as well as the diagnosed mental incapacity. Prior to enrolment, all participants signed an informed consent form. The local ethics committee approved the study protocol.

In order to train and validate the machine learning models, an accurate reference (gold standard) was required. For this reason, the experiments were carried out in the hospital, where it was possible to observe participants closely, perform accurate references and do it under medical and technical supervision.

**Figure 9:**
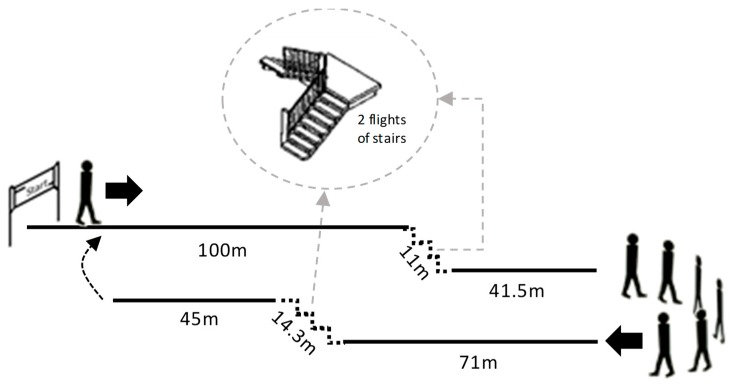
Walk-through circuit designed to acquire data for training and system validation.

During a single visit to the hospital, participants were first interviewed. The Mini-Mental state examination (MMSE) was conducted and dyspnoea was measured before and after the test using the Modified Medical Research Council scale (*mMRC*). Then, subjects were fitted with the designed sensor unit and the Inogen One G2 portable oxygen concentrator. The participants followed the designed study protocol that started with an initial period of three minutes at rest, in a seated position. Next, participants were asked to walk, following the circuit illustrated in Figure 9, for 12 min.

The circuit included sections for walking, climbing and descending stairs. Participants were asked to exercise a gait pattern similar to that maintained in their daily activity in order to capture representative conditions of the activities performed in daily free-living conditions. Along the route, chairs were arranged for the subject to make stops when he/she considered necessary. Throughout the experiment, there were no restrictions on the participant’s body movements. The final activity consisted of sitting for three minutes.

During each test, the stop and start times of each activity were noted and a label was assigned to each period of time. In addition, the number of user interactions with the POC, the flow level selected by the patient in each section of the circuit, the number of rest episodes (seated) of the participant, the oxygen saturation and the heart rate (measured using a Nonin WristOx_2_^®^ Model 3150 pulse-oximeter), and the distance walked were recorded.

### 2.3. Automatic Classification of the Intensity of Physical Activity

#### 2.3.1. Daily Activities in Patients with COPD

The metabolic equivalent of tasks (MET) expresses the energy cost of physical activities and is defined as the ratio of the work metabolic rate to the resting metabolic rate, expressed in kcal/kg/hour. One MET is considered to be equivalent to the energy cost during quiet sitting. In addition, a MET can also be defined as the amount of oxygen consumed while sitting at rest, measured in ml/kg/min. In this case, one MET is equal to the oxygen cost of sitting quietly, equivalent to 3.5 mL/kg/min. A third definition relates MET to the rate of energy produced per unit surface area of an average person seated at rest, expressed in W/m^2^ [45].

Different intensity physical activities entail different energy costs and therefore require varying levels of oxygen consumption. Table 4 describes the physical activities considered in this study. High intensity physical activities were not addressed since they are not expected in patients with COPD and respiratory failure. As described above, the amount of oxygen supplement required for each patient according to the intensity of the physical activity is titrated by the specialist at the initiation of therapy. A change in the intensity of the physical activity performed by the patient poses a need for updating the amount of oxygen provided by the POC [46].

#### 2.3.2. Data Processing and Features Extraction

The data from the accelerometer and from the gyroscope (X, Y and Z axes) were collected at a sampling rate of 25 Hz. The acceleration signals were divided into windows of 3 s (75 samples) without overlap, which was considered enough to capture significant properties of the signal. Activities of different intensity were identified from annotated labels.

In order to remove the DC component in the IMU signals, a high-pass filter with a cut-off frequency of 0.15 Hz was applied. The filtering was implemented using a low-pass IIR filter whose output was subtracted from the original signal.

For each of the time windows, 98 time-domain features were extracted from the six filtered acceleration and gyroscope signals. The explored features are detailed in Table 5. Features from the frequency and non-linear domains were excluded to reduce the computational burden in the microprocessor when implementing the real-time classification model.

#### 2.3.3. Features Selection

**Figure 10:**
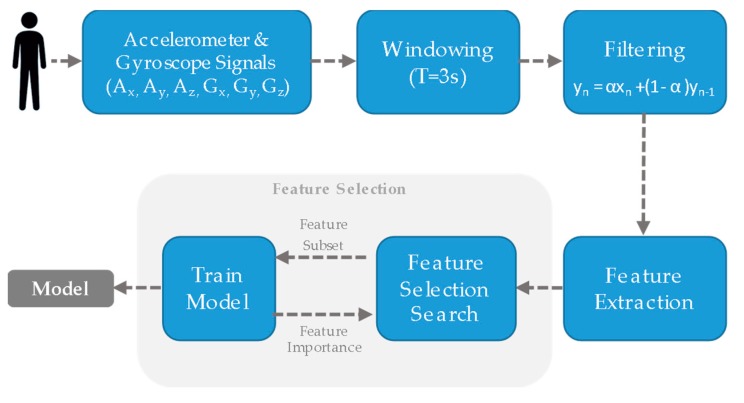
Wrapper approach to feature subset selection. A search algorithm was used through the space of possible features and evaluate each subset by training and cross-validation a model.

In order to improve the computational efficiency and reduce the generalization error of the model by removing irrelevant features, a wrapper feature selection approach was followed. The space of attribute subsets was searched by greedy hill-climbing augmented with a backtracking facility [47]. The search started with an empty set of attributes and search forward. The number of consecutive non-improving nodes allowed before terminating the search was 5. The feature selection algorithm operated in tandem with different machine learning classifiers and classification accuracy at each step was compared. The entire training-internal validation process is shown in Figure 10.

#### 2.3.4. Classification and Internal Validation

**Figure 11:**
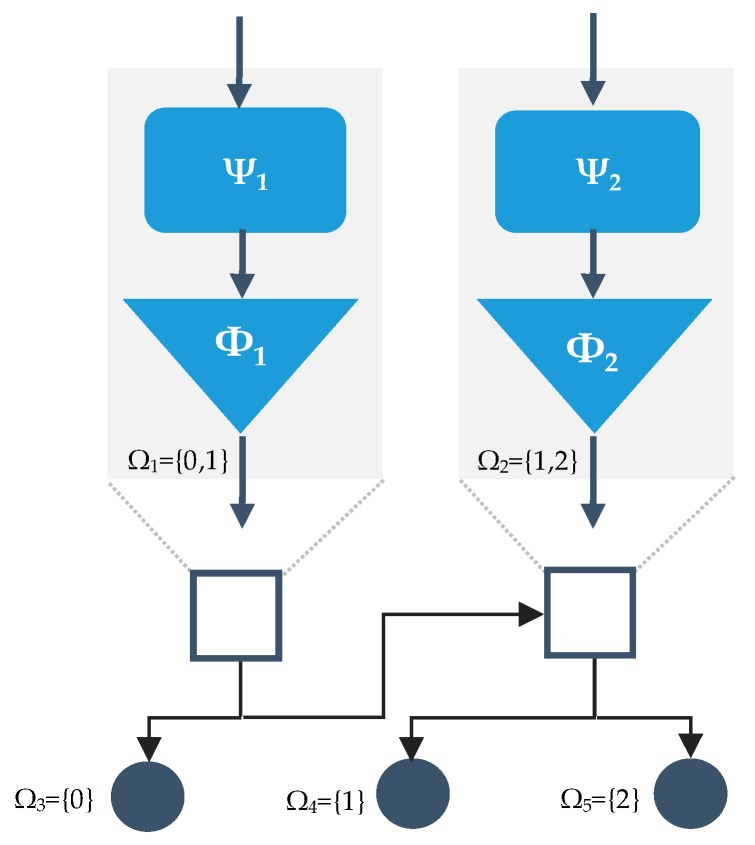
The proposed binary hierarchical classifier (BHC) applied to the classification of the intensity of physical activity. Given that the classifier operates with three classes, its structure has 2 internal nodes and 3 leaf nodes. Each internal node is comprised of a set of features (ψ_i_) and a classifier (ϕi). Each node *n* is associated with a set of classes. In this study, classes are defined as: 0 = sedentary physical activity; 1 = light-intensity physical activity; and 3 = moderate-intensity physical activity.

A binary hierarchical classification structure (BHC) with two classifiers was chosen due to its computational simplicity [48]. Figure 11 shows the proposed architecture of a hierarchical classifier that requires two pairwise classifiers arranged as a binary tree with three leaf nodes, one for each class, and two internal nodes, each with its own feature space. Each of the two internal nodes consisted of a classifier and a set of features specific to it. The coarse separation between classes (sedentary vs active intensity of physical activity) occurred at the upper level in the hierarchy and a finer classification decision (light vs moderate intensity) at a lower level [49]. The architecture had a balanced binary hierarchical structure, in which the two meta-classes at each node had the same number of classes. For this study, decision trees (DT), Linear Discriminant Analysis (LDA), Logistic Regression Classifier (LR), Support Vector Machines (SVM), and Radial Basis Function (RBF) classifiers were evaluated as candidates for each of the internal nodes.

The FSM machine described in Section 2.1.1 was added to the BHC as an output layer to enhance safety. Each classifier was trained with leave-one-subject-out cross-validation (LOSO-CV) scheme, where data from 17 participants were used for training the classifier, and the remaining participant data for evaluating the model performance. This process was repeated 18 times so that each participant was used once for validation.

Several weighted metrics were used to measure model performance: precision, recall, and F1-measure, defined as the weighted average of precision and recall. Additionally, sensitivity (Se), specificity (Sp), and the geometric mean of sensitivity and specificity (G) were estimated. Finally, the receiver operating characteristic (ROC) curve was computed and the area under the curve (AUC) for each class was estimated. Signal processing and model training and validation were performed using MATLAB software (Mathworks Inc., Natick, MA, USA) and DTREG predictive modelling software.

### 2.4. Technical Pilot Test

In the final stage, five participants went through the circuit designed during the previous phase, using the POC in automatic mode (iPOC). Demographic and clinical data for these patients are shown in Table 6.

This feasibility pilot was conducted with the purpose of examining technical and usability issues, and the differences in the changes in blood oxygen saturation with respect to the circuit performed in manual mode. Special attention was paid to the number of oxygen desaturation episodes and to the maximum and minimum SpO_2_ values during the test.

### 2.5. Usability

After each experiment, a semi-structured interview was conducted with the involved patient, in order to obtain information regarding the usefulness, ease of use and expectations of the participant.

In addition to the 18 patients enrolled, 15 additional patients under LTOT were interviewed by phone, in order to have a more significant sample.

Three main questions were asked in the interview:

1. Do you consider a system such as the one proposed to be necessary?
2. Would the automatic concentrator promote your out-of-home activities?
3. In your daily use of the concentrator, do you forget to adjust the recommended dose of O_2_ when the intensity of your physical activity varies?

In questions 1 and 2, the subject was asked to describe his/her degree of agreement on a Likert scale from 1 through to 5, with the strongest positive agreement being 5. To assess usability, the System Usability Scale (SUS) was used in the technical pilot test. SUS is a 10-item questionnaire with 5 response options ranging from strong agreement to strong disagreement. The SUS possible values range from 0 to 100 [50].

## 3. Results and Discussion

### 3.1. Model Training and Internal Validation

**Figure 12:**
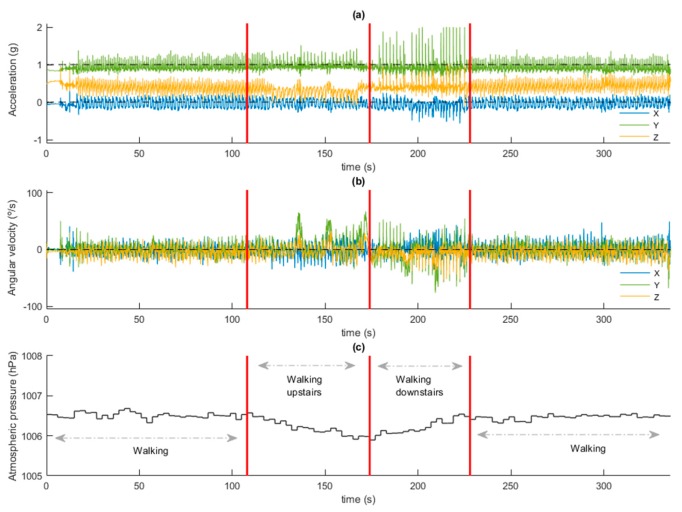
Signals acquired during one of the experiments in the hospital, with the patient walking the circuit designed for the physical test. (**a**) 3-axis accelerometer signals; (**b**) 3-axis gyroscope signals; (**c**) barometer signal. Red vertical lines indicate changes in activity (walking on level, going upstairs, going downstairs).

The dataset used in this study phase included 18 patients, and three different classes with 2160 epochs of sedentary, 3115 epochs of light activity and 550 epochs of moderate activity. Figure 12 illustrates the signals acquired in a time window during different activities along with a test.

Table 7 shows the dimensions of the feature set that resulted from the wrapper-based Sequential Feature Selection (SFS) approach for each of the explored models and classifiers.

Table 8 shows the model performance achieved by classifiers ϕ_1_ and ϕ_2_, respectively, using the selected feature set for each trained and validated algorithm. In the case of ϕ_2_, and given the large imbalance between classes, instances of light activity were weighted in order to balance target categories. The weights were adjusted so that the sum of the weights for the instances within each target category was the same.

The results show that the ϕ_1_ classifier implemented using SVM was able to discriminate between sedentary and active physical activity with a maximum value of F1-measure, G, and AUC of 97.92%, 97.03% and 0.99 respectively. In the case of the ϕ_2_ classifier, designed to discriminate between light and moderate-intensity physical activities, maximum F1-measure, G and AUC were 79.25% (LDA), 97.13% (SVM) and 0.99 (SVM, LDA, MLP) respectively. The lower performance shown by classifier ϕ_2_ is explained by the difficulty in discriminating between up and downstairs activities, which has already been reported in other studies.

According to the results found, the LDA model, which demonstrated a better compromise between sensitivity and specificity, and the higher AUC, was chosen for implementing the classifier ϕ_2_ in the hardware. For classifier ϕ_1_, the J48 decision tree was chosen for the hardware deployment. Although the SVM algorithm obtained better results in terms of G, it was discarded given that the differences in performance were minimal and the computational cost of the SVM and its associated feature space was significantly higher.

**Figure 13:**
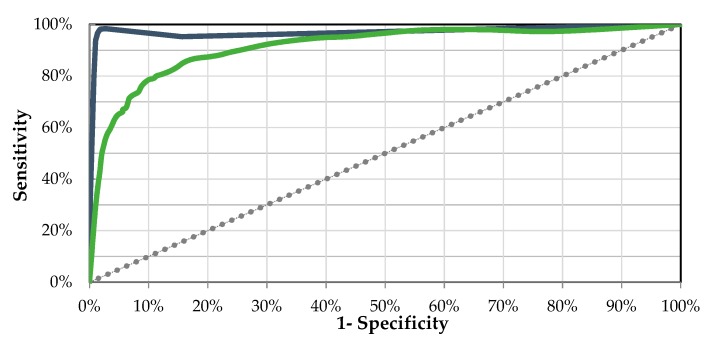
Receiver Operating Characteristics (ROC) Curves for selected models. The blue line represents the ROC curve for the J48 model. The green line shows the ROC curve for the J48 classifier.

Figure 13 shows the Receiver Operation Characteristics (ROC) curves for each class and both classifications approaches.

Table 9 illustrates the confusion matrix and the performance metrics for the build two stages of classification procedure (BHC). The BHC was able to achieve a weighted-F1 measure of 85.9% with weighted-precision and weighted-recall of 85.9%, and 86.1%, respectively.

During the completion of the circuit, the participants had to manually adjust the oxygen flow several times during the route. The total number of adjustments required for the rounds completed was 96. By contrast, participants only manipulated the oxygen concentrator to suit the needs of O_2_ demanded by the physical activity performed on 17 occasions, which showed underuse (only 17.7% of needed adjustments were carried out) of the device that could potentially lead to oxygen desaturation events.

### 3.2. Technical Pilot Test

The technical pilot test was carried out in a controlled environment in the hospital. The participants walked the same circuit designed for data collection for model training and internal validation.

**Figure 14:**
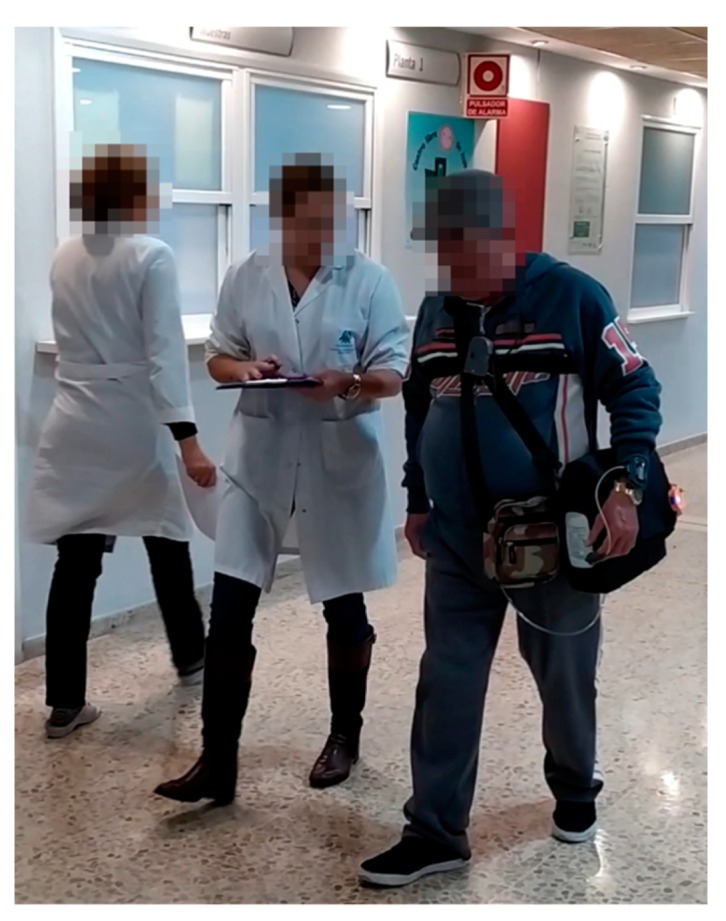
A participant using the automatic portable oxygen concentrator during the technical pilot test.

Figure 14 shows one of the participants during the test carrying the POC and the auxiliary units designed for its automation.

Table 10 synthesizes the results obtained by the BHC using the same performance metrics applied for internal validation.

Again, the accuracy achieved is high (91.1%) but conditioned by the errors in the classification of the activities up/downstairs. These errors are caused by the inherent difficulty in classifying these tasks, and by the different pattern in each patient’s gait, which depends on their physical condition. As a general rule, patients with COPD and respiratory failure who are receiving LTOT avoid using long flights of stairs, thus errors made in this regard cannot be considered critical in a real-life scenario.

Table 11 shows a comparison of the results for changes in blood oxygen saturation for each of the five participants during manual mode (conventional use of the device) and automatic mode tests (proposed iPOC).

An overall reduction in the number of desaturation events can be appreciated in four out of the five subjects. In the same four patients, the average blood oxygen saturation values were increased with respect to the POC test in manual mode. In the same way, the minimum blood saturation values were also improved. The cumulative time spent with SpO_2_ below 90% (CT90) and 85% (CT85) again showed a generally better response when the iPOC was used.

Subject 5 deserves a special mention. He went around the circuit with reduced mobility due to the use of a crutch as a result of an episode of low back pain. Additionally, he showed signs of exacerbation within few days after the test. Some authors have detected significantly decreased saturation in the period of seven days preceding exacerbation [51], so we think that the result of the test could have been biased by these factors.

**Figure 15:**
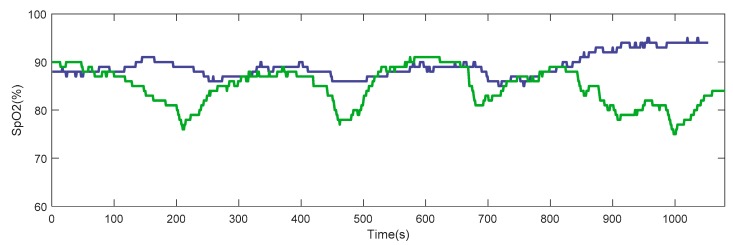
SpO_2_ patterns of a participant during the technical pilot test. The blue line refers to SpO_2_ values while using the portable oxygen concentrator with the proposed automatic mode. The green line refers to SpO_2_ values using the POC in manual (conventional) mode.

Figure 15 illustrates the change in blood oxygen saturation pattern on exertion for one of the participants. The stabilization of SpO_2_ values, the reduction in the number of desaturation events, and the higher mean value obtained with the use of the proposed system can be clearly observed.

Improvement in oxygenation is attributable to the fact that the adjustment of oxygen flow during daily living activities in response to higher oxygen demand is done, in conventional POCs, manually by the patient. Incorrect use in the oxygen flow adjustment or adjustment made with a delay relative to the change in physical activity intensity often leads to desaturation episodes [24].

### 3.3. Usability

Table 12 presents a synthesis of the results obtained in the semi-structured interview with the participants in the initial phase.

All the patients interviewed considered that a system capable of automatically regulating the oxygen flow in the concentrator was necessary. They also felt that such a system would help increase physical activity outside the home. The high percentage of patients who admit to forgetting to adjust the concentrator when faced with changes in physical activity intensity (51.5%) is noteworthy. Similarly, the percentage of patients who consciously skip the adjustment of the oxygen flow is outstanding. Finally, the average SUS score calculated after the technical pilot test was 79 ± 11.4. There was one participant that had an average SUS score of 95, followed by scores of 85 (1/5), 75 (2/5), and 65 (1/5). In the research, the average SUS value is 68, which can be considered a benchmark. The achieved value of 79 indicates significantly better usability than average [52].

## 4. Conclusions

The long-term benefits of oxygen have been proven since the 1980s in certain respiratory conditions such as COPD. Very recently, future research in oxygen therapy has been pointed at developing and evaluating new models for therapeutic oxygen patient education and improving portable oxygen devices [53]. It has been reported that, when compared to the conventional POCs, the closed-loop POCs can maintain higher saturation levels, spend less time below the target saturation, and save O_2_ resources [25]. The correction of exercise hypoxemia in lung diseases like COPD is crucial and challenging [54], and automatic POCs can contribute to the individualized adjustment of oxygen flow.

Currently, the challenge of designing closed-loop portable devices which are able to adjust O_2_ flow automatically is being faced by mainly using indirectly measured blood oxygen saturation as a process variable to close the loop. However, the robustness of control algorithms and the limited reliability of current oximetry sensors are hindering the effectiveness of this approach.

In line with all the above-mentioned factors, this study presents the proof of concept of an alternative approach: a system to transform a conventional POC into a closed-loop controlled device capable of automatically self-adjusting, in real time, the oxygen flow supplied to the patient according to the intensity of the physical activity carried out by the user under LTOT.

A sensor unit capable of detecting the physical intensity developed by the patient (in real time) has been developed and evaluated. This sensor unit can wirelessly connect to the POC for the self-adjustment of the oxygen flow. The system has been designed with the flexibility to customize up to seven different oxygen therapy profiles. The developed iPOC was tested with a widely used commercial POC unit. For this purpose, a treadmill circuit was designed that included basic physical activities most common in the daily practice of elderly patients with respiratory disorders. Different models were trained and validated using artificial intelligence techniques, a wrapper approach, 98 time-domain features, and data from 18 patients with COPD and respiratory failure. A final meta-classifier (BHC) was designed and deployed in the sensor unit to operate in real-time. A weighted accuracy of 91.1% was achieved in the technical pilot test with five patients. A reduction in the number of desaturation events was achieved in 80% of patients as well as improved minimum and average blood oxygen saturation values compared to the POC in manual operating mode. In these cases, CT90 and CT85 also showed a promising better response when the iPOC was used. Finally, all interviewed patients (N = 33) considered that the proposed iPOC satisfied them, and could promote their physical activity outside the home.

Among the limitations of this study were those related to hypoxia. In this regard, the combination of the proposed system with the novel oxygen reserve index (ORI) included in the new generation of pulse oximeters that use multi-wavelength pulse co-oximetry might improve oxygen titration and enable the prevention of unintended hyperoxia [55].

A closed-loop control system, like the proposed iPOC, has clear potential benefits, including improved oxygenation regardless of physical activity and enhanced patient follow-up and compliance with therapy recommendations. In addition, closed-loop oxygen supply systems have shown that they can potentially reduce medical error, improve morbidity and mortality, and reduce care costs [22].

Future research steps include the miniaturization of the sensor unit, expanding the study sample, and the home monitoring of the patients while using IPOC during daily tasks in an unsupervised environment, in order to obtain clinical evidence of the impact that this approach may have on the targeted patient population.

## 5. Patents

DSM and ALJ are the inventors of the utility model ES U201831680 with the title ‘Automatic flow-metering device for oxygen therapy equipment’.

## Figures and Tables

**Table 1:** Personalisation of oxygen therapy in the control unit. Seven different options were available. Bolus volume (pulse setting 1 to 5) delivered to patient depending on the intensity of physical activity ranges from 180 mL (pulse setting 1) to 900 mL (pulse setting 5).

Setting	Pulse Setting according to Physical Activity			Jumpers Setting		
	Sedentary	Light	Moderate	P3	P2	P1
1	1	2	4			X
2	1	3	5		X	
3	1	4	5		X	X
4	2	4	5	X		
5	3	4	5	X		X
6	0	2	5	X	X	
7	0	1	4	X	X	X

**Table 2:** Bolus volumes delivered by Inogen One G2 at 20 °C at sea level at different breathing frequencies.

Pulse Setting	Pulse Volume (ml ± 10%)		
	15 Breaths per Minute	20 Breaths per Minute	25 Breaths per Minute
1	12	9	7.2
2	24	18	14.4
3	36	27	21.6
4	48	36	28.8
5	60	45	36.0

**Table 3:** Demographic and clinical data for patients in the internal validation group.

Characteristics	Data *
N	18
Male/Female	14/4 (77.7%/22.3%)
Age (years)	66.9 ± 12.8 (60–93)
51–60	5.6% (1)
61–70	44.4% (8)
71–80	16.7% (3)
81–90	33.3% (6)
BMI (Kg/cm^2^)	27.0 ± 3.6
LTOT history (months)	22.1 ± 18.1
POC history (months)	19.5 ± 13.2
Daily hours using the POC	2.3 ± 1.0
mMRC dyspnoea baseline level	2.6 ± 0.5
FEV1/FVC	0.5 ± 0.2

*BMI*: Body mass index; *LTOT*: long term oxygen therapy; *POC*: portable oxygen concentrator; *mMRC*: Modified Medical Research Council scale; *FEV_1_*/*FVC*: forced expiratory volume in 1-s to forced vital capacity ratio. * Results expressed as mean ± SD, except where otherwise indicated.

**Table 4:** Activities, grouped by intensity, associated MET, and the number of instances.

Intensity	Activity	METs (W/kg)	Number of Instances
Sedentary	Sitting Standing Lying	1.3	2160
Light	Walking	2	3115
Moderate	Walking upstairs Walking downstairs	3.5–5	550
**Total**			**5825**

*MET*: The metabolic equivalent of task.

**Table 5:** Initial feature set.

Feature Set (Accelerometer + Gyroscope)	Features
RMS, standard deviation, absolute mean	18
Mean of the derivative	6
Pairwise correlations	6
Simplified energy	6
Moments (skewness, kurtosis, median)	18
Min, max, difference between max and min	18
Interquartile range	6
Signal magnitude vector (mean, standard deviation, median, skewness, kurtosis)	10
Signal magnitude vector (interquartile range, max, min, the difference between max and min)	8
Signal magnitude area	2
**Total**	**98**

*RMS*: Root mean squared.

**Table 6:** Demographic and clinical data for patients in the technical pilot test.

Characteristics	Data *
N	5
Male	5 (100 %)
Age (years)	72.2 ± 6.5
BMI (Kg/cm^2^)	28.4 ± 6.0
LTOT history (months)	27.6 ± 25.7
POC history (months)	22.8 ± 15.5
Daily hours using the POC	4.2 ± 2.4
mMRC (baseline dyspnoea index)	2.6 ± 0.5
FEV_1_/FVC	0.5 ± 0.2

*BMI*: Body mass index; *LTOT*: long term oxygen therapy; *POC*: portable oxygen concentrator; *mMRC*: Modified Medical Research Council scale; *FEV_1_*/*FVC*: forced expiratory volume in 1-s to forced vital capacity ratio. * Results expressed as mean ± SD, except where otherwise indicated.

**Table 7:** The optimal feature set obtained from Wrapper-based feature selection for the classifiers ϕ_1_ and ϕ_2_.

Method	Selected Features for ϕ_1_	Selected Features for ϕ_2_
LR	8	17
J48	4	14
SVM	15	14
LDA	9	20
MLP	11	12

*LR*: logistic regression; *J48*: C4.5 decision tree; *SVM*: support vector machines; *LDA*: linear discriminant analysis; *MLP*: multilayer perceptron.

**Table 8:** Performance metrics of classifiers ϕ_1_ and ϕ_2_ for each cross-validated machine learning algorithm.

Method	Accuracy		F1-Measure		Se		Sp		*G*		AUC	
	ϕ_1_	ϕ_2_	ϕ_1_	ϕ_2_	ϕ_1_	ϕ_2_	ϕ_1_	ϕ_2_	ϕ_1_	ϕ_2_	ϕ_1_	ϕ_2_
LR	94.54	91.86	95.60	64.71	95.70	51.16	93.38	98.81	94.53	71.10	0.98	0.85
J48	96.45	74.72	96.98	74.15	96.76	72.55	95.97	76.89	96.36	74.69	0.98	0.74
SVM	97.13	88.49	97.92	61.18	97.44	35.82	96.62	97.78	97.03	59.18	0.99	0.81
LDA	95.30	84.01	95.93	79.25	93.87	75.09	97.36	85.59	95.60	80.17	0.99	0.88
MLP	96.02	77.24	96.62	77.58	96.31	78.73	95.60	75.76	95.95	77.23	0.99	0.85

*LR*: logistic regression; *J48*: C4.5 decision tree; *SVM*: support vector machines; *LDA*: linear discriminant analysis; *MLP*: multilayer perceptron; *Se*: Sensitivity; *Sp*: specificity; *G*: geometric mean of sensitivity and specificity; *AUC*: area under the receiver operating characteristic curve (*ROC*) curve.

**Table 9:** Performance metrics for the binary hierarchical classification structure for each of the levels of intensity of physical activity.

		Predicted		Performance			
**True**		*S*	*Other*	**Precision**	**Recall**	**Accuracy**	**F1-Measure**
	*S*	2073	77	95.4%	96.4%	97.0%	95.9%
	*Other*	101	3666				
		*L*	*Other*				
	*L*	1569	335	76.1%	82.4%	85.9%	79.1%
	*Other*	493	3466				
		*M*	*Other*				
	*M*	1393	470	82.9%	74.8%	86.9%	78.6%
	*Other*	288	3642				

*S*: Sedentary; *L*: light intensity of physical activity; *M*: moderate intensity of physical activity.

**Table 10:** Weighted performance metrics of the binary hierarchical classification structure in the technical pilot test.

Accuracy	F1-Measure	Precision	Recall
91.1%	76.6%	74.9%	83.6%

**Table 11:** Changes in blood oxygen saturation levels during the tests performed using the conventional portable oxygen concentrator (POC) and the proposed intelligent POC (*iPOC*).

Mode	Automatic POC (iPOC)					Manual POC				
Subject	*S1*	*S2*	*S3*	*S4*	*S5*	*S1*	*S2*	*S3*	*S4*	*S5*
SpO_2_ events	2	1	1	2	3	3	3	6	1	2
Mean SpO_2_	89.5	92.2	89.1	94.7	87.1	88.6	90.4	86.1	91.5	89.5
min (SpO_2_)	77.0	87.0	85.0	89.0	81.0	80.0	86.0.	75.0	81.0	93.0
CT90	50.1	18.8	70.9	8.4	73	62.9	33.1	91.7	20.9	60.6
CT85	34.9	0.0	0.0	0.0	27.6	17.4	0.0	33.9	2.8	6.3

*CTxx*: time spent with SpO_2_ < xx%.

**Table 12:** Results from the semi-structured interview.

Question	Average Likert Score
1. Do you consider a system such as the one proposed to be necessary?	5 ± 0 (33)
2. Would you the automatic concentrator promote your out-of-home activities?	5 ± 0 (33)
3. In your daily use of the concentrator, did you forget to adjust the recommended dose of O2 when your degree of physical activity varies?	
Yes The patient consciously does not adapt the flow The patient ignores that he has to adjust it	51.5% (17) 30.3% (10) 18.2% (6)
